# Study on Zoledronic Acid Reducing Acute Bone Loss and Fracture Rates in Elderly Postoperative Patients with Intertrochanteric Fractures

**DOI:** 10.1111/os.12460

**Published:** 2019-05-06

**Authors:** Zhong Liu, Chun‐wen Li, Yi‐fan Mao, Kang Liu, Bo‐cheng Liang, Lian‐guo Wu, Xiao‐lin Shi

**Affiliations:** ^1^ The Second Clinical Medical College Zhejiang Chinese Medical University Hangzhou China; ^2^ Department of Diagnostics of Traditional Chinese Medicine, College of Basic Medical Science Zhejiang Chinese Medical University Hangzhou China; ^3^ Department of Osteology The Second Affiliated Hospital of Zhejiang Chinese Medical University Hangzhou China

**Keywords:** Bone loss, Intertrochanteric fracture, Osteoporosis, Zoledronic acid

## Abstract

**Objective:**

To observe the effect of zoledronic acid on the reduction of acute bone loss and fracture rate in elderly postoperative patients with intertrochanteric fracture.

**Methods:**

From August 2012 to January 2015, a total of 482 patients with senile osteoporotic femoral intertrochanteric fracture, who accepted proximal femoral intramedullary fixation under anesthesia were analysed. The patients were divided into two groups. Treatment group (353 cases) were treated with 100 mL/5 mg of zoledronic acid injection in 1 week after operation, as well as orally taken 600 mg/d of calcium carbonate and active vitamin D3 400 IU/d. Control group (129 cases) were given the same dose of calcium carbonate and active vitamin D3 orally. Efficacy evaluation were conducted during different periods of medication

**Results:**

Compared with pre‐medication, indexes of bone metabolism (TARP‐5b, CTX) in the treatment group were brought down, especially significantly statistically different after 12 months of medication. The treatment group performed superior to control group in alleviating the pain of back and posture changing (*P* < 0.05), improving bone density (*P* < 0.05), depressing re‐fracture rate (*P* < 0.01) after 24 months of medication. In addition, BP, PF and MH dimension scores were demonstrated with statistical significance (*P* < 0.05).

**Conclusions:**

The application of zoledronic acidin elderly postoperative patients with intertrochanteric fracture can not only relieve acute bone loss, reduce the incidence rate of re‐fracture, alleviate osteoporosis pain and the pain from osteoporotic fracture, but also improve bone metabolism and quality of life, which may offer an acceptable clinical opinion

## Introduction

Primary osteoporosis, a systemic chronic metabolic disease characterized by reduced bone quality and bone mass, occurs frequently in menopausal and postmenopausal women. The most serious consequence of osteoporosis is brittle fractures caused by low energy damage, which are associated with a high mortality rate and have a serious influence on the quality of life of patients with osteoporosis. In view of the serious consequences of osteoporosis, many preventive and therapeutic drugs have appeared in clinical application^1^, ^2^. Drugs currently available for usage in elderly postoperative patients with intertrochanteric fractures are limited or flawed. According to NOF in the USA, effective drugs approved by the Food and Drug Administration should be applied in addition to improving lifestyle, supplying vitamin D and calcium, and including drugs that inhibit bone resorption (i–iii) and bone‐promoting drugs (iv): (i) diphosphonates (zoledronic acid, alendronate, preparation of alendronate with vitamin D, ibandronate, risedronate, and 500‐mg calcium carbonate preparations of risedronate); (ii) calcitonin; (iii) estrogen or reynoxifene; and (iv) parathormone [PTH(1‐34)]. Zoledronic acid is one of the most commonly used drugs for the treatment of osteoporosis in clinical application as the third generation of bisphosphonate medicine containing imidazole heterocycle. Zoledronic acid inhibits the action of FPP synthase, promotes osteoclast apoptosis, and inhibits bone resorption. To further clarify the therapeutic effect of zoledronic acid on reducing acute bone loss and fracture incidence in elderly postoperative patients with intertrochanteric fractures, we collected 353 cases of senile osteoporotic femoral intertrochanteric fractures, among patients who were treated with zoledronic acid in the early postoperation period. The clinical observations and curative effects were evaluated, including the reduced acute bone loss, the recurrence rate of fractures, pain, the index of bone metabolism, and the quality of life.

## Overall Design and Follow‐up Method

### *General Data and Treatment Methods*

From August 2012 to January 2015, 482 patients with senile osteoporotic femoral intertrochanteric fractures in the Department of Orthopedics at The Second Affiliated Hospital of Zhejiang Chinese Medical University were treated with intramedullary fixation of the proximal femur under anesthesia. Simple random grouping was used to divide patients into two groups. The 353 patients in the treatment group, including 189 men and 164 women who were 60–95 years old (Table 1), were medicated within 1 week after the fracture, with an intravenous drip of 100 mL/5 mg zoledronic acid injection, infusion time > 30 min, supplemented with 500 mL normal saline, 600 mg/d calcium carbonate taken orally, and active vitamin D3 400 IU/d. The 129 patients in the control group were given the same dose of calcium carbonate and active vitamin D3 orally. The groups were evaluated before medication, and after 1 week of medication, 12 months of medication, and 24 months of medication, respectively.

**Table 1:** Comparison of the general condition between the treatment group and the control group

Demographics	Treatment group	Control group	*P*‐value
Cases	353	129	—
Age (years)	75.41 ± 12.54	73.25 ± 13.75	0.104
Age of menarche (years)	14.82 ± 3.26	14.47 ± 3.58	0.31
Age of menopause (years)	49.94 ± 8.64	49.11 ± 7.22	0.33
Height (cm)	163.12 ± 26.62	165.82 ± 25.61	0.32
Weight (kg)	62.44 ± 12.11	64.13 ± 15.67	0.212
Body mass index (kg/m^2^)	23.54 ± 8.24	22.26 ± 9.55	0.149
Past fracture history (years)	35	16	0.384
Smoking history (years)	48	17	0.072

Note: After the *t*‐test and χ^2^‐test, there was no significant difference between the treatment group and the control group (*P* > 0.05). There is comparability

#### *Inclusion Criteria*

Inclusion criteria include: (i) bone mineral density value determined by dual energy X ray T < −2.5 or T < − 1.0 and past history of brittle fracture; (ii) no serious liver and renal dysfunction; (iii) no caltrate and calciferol allergy history, confirmed and with informed consent provided for diagnosis and treatment; and (iv) the occurrence of senile osteoporotic femoral intervertebral fracture with low energy fracture, 2 weeks after surgical treatment.

#### *Exclusion Criteria*

Exclusion criteria include: (i) past history: (3 months before treatment) used estrogen, glucocorticoid, and other drugs that may affect bone metabolism; (ii) there were thyroid or parathyroid diseases, or adrenal and gonadal endocrine diseases; (iii) secondary osteoporosis; (iv) patients allergic to zoledronic acid or inability to use zoledronic acid; (v) patients with serious Alzheimer's disease are not suitable for treatment and evaluation of therapeutic effects; and (vi) severe hypocalcemia.

**Figure 1:**
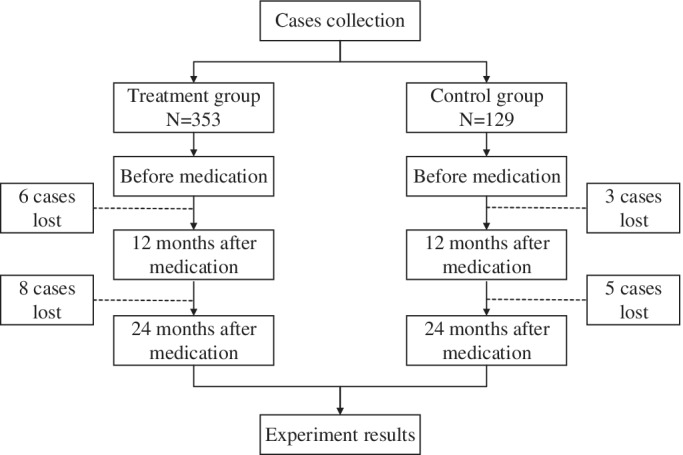
Cases collection and experiment results.

An overview of cases collection is shown in Fig. 1.

### *Index of Effect Evaluation*

#### *Pain*

The visual analog scale (VAS) scoring standard was used to evaluate the pain level of the patients. The VAS pain scoring standard (scores from 0 to 10) was as follows: 0 = painless; less than 3 = mild pain that the patient can endure; 4–6 = patient was in pain but can sleep and pain could be endured; and 7–10 = patient had intense pain and was unable to tolerate the pain. After the treatment, the effect was obvious if the score was reduced by more than 4, and decreased by 2 to 3. Once the score had decreased by less than 2 was invalid. The total effective rate = obvious effect + effect/total cases × 100%. Physical examination was carried out before medication, pain of the knee joint, back pain, and postural pain were recorded.

#### *Bone Mineral Density*

Dual energy X‐ray absorptiometry was used. Measurement location: lumbar vetebra L_2–4_. The fracture healing time and the incidence of refractures were compared between the two groups during the follow‐up period.

#### *Index of Bone Metabolism*

Blood sample collection: The blood samples and numbers of the subjects were indicated in advance on the blood tube. All patients had an empty stomach for 8–12 h before the blood was drawn and the blood was taken using standard vein blood sampling.

Blood samples were centrifuged; blood samples were collected and centrifuged under 3000 *g* for 10 minutes within 3 h after blood collection.

The serum was immediately transferred to the EP tube. (If there is a delay, the sample should be kept at 4°C.)

Serum separation and cryopreservation: All samples were frozen and stored at −80°C.

The level of bone alkaline phosphatase (BALP) was measured using a BPC semiautomatic biochemical analyzer. The serum bone gla protein (BGP) and type I collagen crosslinked C‐terminal peptide (CTX) were measured by radioimmunoassay, and the anti‐tartaric acid phosphatase 5b (TRAP‐5b) was measured by ELISA.

#### *Life Quality Score*

We used a concise health survey scale (SF‐36): the table was developed by the Boston Institute of Health^3^, including 11 items reflecting the two aspects of physical health (PH) and mental health (MH), which were mainly composed of 8 dimensions: general health (GH), body pain (BP), physiological function (PF), vitality (VT), role physiological (RP), role emotional (RE), mental health (MH), and social function (SF). The higher the score, the better the life quality would be.

### *Statistical Method*

The data were processed by SPSS 22.0 software and the measurement data were expressed by mean ± standard deviation (mean ± standard deviation). The paired *t*‐test was used before and after treatment, with *P* < 0.05 as a significant difference, which was statistically significant.

## Result

### *Pain Symptoms and Quality of Life*

After 12 months of medication, the pain score for posture change in the treatment group was lower than that of the control group (*P* < 0.05). After 24 months of treatment, the waist and back pain and posture change pain in the treatment group was better than that of the control group (*P* < 0.05), and the total effective rate of the treatment group was 88.91%, which was significantly higher than that of the control group 80.31% (*P* < 0.05), as summarized in Table 2.

**Table 2:** Comparison of pain scores of knee joint, waist and back and posture change between the treatment group and the control group before and after medication

Type of pain	Treatment group				Control group			
	cases	Before medication	12 months	24 months	cases	Before medication	12 months	24 months
Knee joint pain	69	5.92 ± 0.13	2.42 ± 0.29	1.28 ± 0.63	38	5.88 ± 0.32	2.47 ± 0.79	1.32 ± 0.49
Waist and back pain	124	4.82 ± 0.34	2.39 ± 0.69	1.32 ± 0.51^*^	32	4.92 ± 0.97	2.39 ± 0.33	1.40 ± 0.76^†^
Posture change pain	332	7.12 ± 0.21	3.36 ± 0.42^*^	1.22 ± 0.67^*^	109	7.18 ± 0.36	3.42 ± 0.76	1.28 ± 0.35^†^
Total effective rate %			88.91				80.31	

Note: *At 12 months compared with 24 months in the group (*P* < 0.05), there are significant difference and statistical significance; ^†^two groups in the same period are compared (*P* < 0.05) and there are significant difference and statistical significance. GH, general health; BP, body pain; PF, physiological function; VT, vitality; RP, role physiological; RE, role emotional; MH, mental health; SF, social function

### *Fractures*

Table 3 illustrates that after 12 months of treatment, the bone density of the treatment group was not significantly different than that before treatment. After 24 months, the improvement in bone density in the treatment group was better than that in the control group. There was a significant difference between the groups (*P* < 0.05), and the rate of refracture in the treatment group was lower than that of the control group (*P* < 0.01).

**Table 3:** Comparison of bone mineral density in two groups before and after medication

Groups	Bone mineral density (kg/m^2^)			Refracture rate	
	Before medication	12 months	24 months	(Cases)	Incidence (%)
Treatment group	0.70 ± 0.06	0.81 ± 0.05	0.85 ± 0.03*	21	5.9
Control group	0.69 ± 0.05	0.71 ± 0.04	0.78 ± 0.09^†^	11	8.5^†^

Note: *At 12 months compared with 24 months in the group (*P* < 0.05), there are significant difference and statistical significance; ^†^two groups in the same period are compared (*P* < 0.05) and there are significant difference and statistical significance

### *Bone Mineral Density and Biochemical Markers*

Based on observation, there was no significant difference between groups before the medication. After 1 week of medication, the indexes of bone metabolism in the treatment group decreased. The CTX and TARP‐5b were statistically different from those before the medication. After 12 months of medication, the two indexes of TARP‐5b and CTX in the treatment group were significantly statistically different from those before the medication. After 24 months of treatment, the BGP, CTX, and TARP‐5b of the treatment group were statistically different compared with those before medication. After 24 months, there was a significant statistical difference between TARP‐5b and CTX in the two groups (*P* < 0.05); see Table 4.

**Table 4:** Comparison of bone metabolism indexes between the treatment group and the control group

Bone metabolism indexes	Before medication		After 1 week		After 12 months		After 24 months	
	Treatment group	Control group	Treatment group	Control group	Treatment group	Control group	Treatment group	Control group
BGP (ng/mL)	12.01 ± 3.28	12.28 ± 3.52	6.13 ± 0.45	8.89 ± 4.01	7.33 ± 1.45	8.18 ± 4.24	7.53 ± 2.25^*^	8.39 ± 2.60
BALP (μg /L)	3.95 ± 1.27	3.32 ± 1.96	3.27 ± 2.91	3.91 ± 1.39	3.44 ± 3.52	3.41 ± 3.56	3.41 ± 2.53	3.24 ± 4.21
CTX (ng/mL)	0.94 ± 3.43	0.91 ± 1.45	0.59 ± 2.19^*^	0.89 ± 1.22	0.69 ± 4.23^*^	0.89 ± 1.40	0.61 ± 1.45^*^	0.96 ± 2.53^†^
TARP‐5b (U/L)	14.34 ± 2.68	10.41 ± 3.15	11.86 ± 1.48^*^	9.92 ± 2.15^†^	12.16 ± 2.53^*^	9.92 ± 2.42^†^	11.25 ± 2.39^*^	10.24 ± 3.18^†^

Note: *Comparing 1 week, 12 months, and 24 months after treatment within the group (*P* < 0.05), there are statistical significance; ^†^two groups in the same period are compared (*P* < 0.05) and there are statistical significance

### *Quality of Life*

There were significant differences in BP, PF, and MH dimension scores in the treatment group after 24 months of medication, with statistical significance (*P* < 0.05). After 24 months of medication, the BP and PF dimension scores were better than those in the control group, with statistical difference (*P* < 0.05); see Table 5.

**Table 5:** Comparison of quality of life scores between the treatment group and the control group

Quality of life	After 12 months		After 24 months	
	Treatment group	Control group	Treatment group	Control group
GH	45.58 ± 8.38	44.24 ± 7.49	54.65 ± 8.69	46.34 ± 9.45
BP	60.32 ± 13.52	59.96 ± 12.95	68.45 ± 14.32^*^	60.79 ± 11.54^†^
PF	50.52 ± 16.55	51.45 ± 17.49^†^	55.43 ± 16.43^*^	50.75 ± 18.52^†^
VT	40.57 ± 8.48	43.58 ± 8.01	58.34 ± 9.29	47.32 ± 8.30
RP	32.60 ± 5.85	31.96 ± 4.29	38.60 ± 5.25	35.38 ± 4.29
RE	49.19 ± 19.28	48.05 ± 18.29	66.30 ± 17.02	54.59 ± 18.31
MH	40.29 ± 12.47	38.86 ± 11.49	55.33 ± 11.01^*^	40.68 ± 14.52
SF	70.48 ± 18.35	66.38 ± 16.93	80.38 ± 18.63	63.58 ± 19.58

Note: *At 12 months compared with 24 months in the group (*P* < 0.05), there are significant difference and statistical significance; ^†^two groups in the same period are compared (*P* < 0.05) and there are significant difference and statistical significance

### *Adverse Events*

Fever, myalgia, shivering, runny nose, nasal congestion, and other influenza‐like symptoms (42.2%) appeared in 149 patients in the treatment group. Zoledronate would inhibit the effect of FPP synthase, promote the activation of T cells, and secrete a large number of cytokines such as interferon‐gamma, tumor necrosis factor‐alpha, and interleukin‐6; it also caused fluctuations in blood calcium, making it easy for patients to become hypocalcemic^4^. Most of the symptoms appeared after 24–72 h of medication. There were 5 cases of arrhythmia (0.01%); all were treated with symptomatic treatment and all symptoms could be relieved reactions. There were no liver and kidney function declines, lesions, rashes, diarrhea, and other adverse effects. During the observation period, in the follow up within 24 months of medication, no serious adverse reactions such as kidney damage, gastrointestinal discomfort, and mandibular necrosis were found. There were 15 cases of gastrointestinal adverse reactions such as heartburn, nausea, and vomiting in the control group. The incidence of adverse reactions was 11.6%, which improved after symptomatic treatment. There was no significant difference in adverse reactions between the two groups (*P* > 0.05).

## Discussion

Osteoporosis is a complex pathophysiological change, mainly manifested as low bone mass, destruction of bone microstructure, increased bone fragility, and predisposition to fracture. Femoral intertrochanteric fractures are the most dangerous type of osteoporotic fractures in the elderly, with high incidence in elderly patients. Most of the patients have other diseases, are at high risk for surgical treatment, and have high disability and mortality rates. Acute bone loss was found in bedridden patients with limb braking (1% of whole body bone mass was reduced as a result of bed‐rest immobilization for 1 week), which causes secondary osteoporosis and affects fracture healing^5^. The possibility of refracture is accordingly increased, thus forming a vicious cycle. Once low energy fragility hip fractures occurred in those over 60 years old, the mortality would be greatly increased in the following 5–10 years. The risk of refracture is 3–5 times higher than that of an average normal person after a fracture heals in an individual with osteoporosis^6^. If a fragility fracture occurs again, the mortality rate would be increased by 3–4 times within 5 years^7^. The usage of zoledronic acid can control bone loss and reduce the risk of refracture, thereby reducing the disability and mortality ratea, improving the quality of life, and prolonging the life of patients.

In recent years, the treatment of osteoporosis has made great progress. The main therapeutic drugs include bisphosphonates, estrogen, and calcitonin. The mechanisms of various drugs are not the same, but most of the drugs achieve the purpose of treating osteoporosis by inhibiting osteoclast‐mediated bone resorption^8^, ^9^, ^10^. Bisphosphonates (BP) are widely used as front‐line drugs for osteoporosis, as well as in the treatment of osteoporosis, bone metastases, and primary bone tumors^11^. Zoledronic acid injection is a third generation bisphosphonates compound, whose unique R side chain containing dinitrogen imidazole heterocyclic structure had a stronger bonding force between the drug and bone surface, as well as resulting in stronger inhibition of osteoclasts^12^. The drugs were absorbed by the bone tissue quickly after injection, directly combined with the bone surface, and entered the osteoclast to inhibit the activity of enzyme FPP synthase necessary for the synthesis of cellular structural proteins in osteoclasts by inhibiting the pathway of mevalonate. It would cause the apoptosis of osteoclasts and inhibition of bone resorption mediated by osteoclasts, to activate the suppressed osteoblasts, correct the imbalance of bone metabolism, and promote bone mass increase^13^. Compared with other bisphosphonates, zoledronic acid has high bioavailability and a longer half‐life period which increases patient compliance after intravenous administration. Osteoporosis is a chronic disease with poor treatment compliance; it is reported that only 42.93% of osteoporosis patients were compliant, mainly due to the use of oral drugs (oral drugs are easy to forget to take and can be associated with gastrointestinal reactions, for instance)^14^. Zoledronic acid has high compliance because it used only once a year. In addition, the unique dinitrogen imidazole heterocyclic structure characteristic of zoledronate sodium makes it bind more to hydroxyapatite on the bone surface^15^.

The main cause of acute bone loss after intertrochanteric fracture is bone atrophy from disuse. With osteoporosis already existing before the operation, the bone mass is rapidly lost due to the bed‐rest and immobilization during the acute period after the fracture. The degree of osteoporosis will be significantly increased after the operation, and the bone mineral density will continue to decline in following 3–6 months^16^. Bone remodeling is a process of coupling new bone formation mediated by bone cells and bone resorption mediated by osteoclasts. In the process of bone tissue reconstruction, bone matrix is constantly generated and decomposed. Specific substances such as lysates, matrix products, and enzymes enter urine or blood be regarded as markers of bone transformation. Bone metabolic markers are associated with bone turnover. The increase of bone turnover markers level will accelerate bone loss and cause bone mineral density to decrease. Through testing it was evident that the bone metabolism index of the treatment group was reduced after 1 week of medication. The CTX and TARP‐5b were statistically different from those before the medication. This showed that Aclasta could reduce the concentration of bone metabolic markers, alleviate the acute bone loss, and improve the bone metabolism of postoperative intertrochanteric fracture patients.

The persistent bone loss is caused by bed rest, immobilization, and other measures after the occurrence of osteoporotic fractures, resulting in a decrease in bone mineral density. To a certain extent, osteoporosis cannot be clinically manifested but can change the patient's body. Bedridden patients, engaging in little movement, would suffer bone mineral loss, resulting in further osteoporosis and increasing the incidence of refracture, which would cause serious threat to the patients’ lives^17^. According to this study, all bone metabolism indexes decreased after 1 week of treatment; the CTX and TARP‐5b were statistically different from those before the medication. After 12 months of medication, these two indexes in the treatment group were significantly statistically different from those before the medication. The improvement in bone mineral density in the treatment group was more obvious than that of the control group after 24 months of medication, with statistical significance (*P* < 0.05); also, the incidence of recurrence of intertrochanteric fractures in the treatment group was significantly lower than in the control group, with statistical significance (*P* < 0.01); the BGP, CTX, and TARP‐5b indexes were significantly statistically different from those before the medication. The BP, PF, and MH dimension scores of the treatment group were statistically significant (*P* < 0.05) between 12 and 24 months of medication. The treatment group performed better than the control group in BP and PF dimension scores after 24 months of medication, with statistical significance (*P* < 0.05). Therefore, zoledronic acid is of great significance in reducing the risk of fracture and acute bone loss. In addition, zoledronic acid has been proved as a safe and reliable drug with no severe adverse drug reactions, based on 6–8 years of clinical reports currently with 149 patients in this treatment group after 24 months of medication.

Above all, the application of zoledronic acid injection after surgery for osteoporotic femoral intertrochanteric fracture can obviously relieve the acute bone loss due to fracture, reduce the incidence of refracture, relieve the pain caused by osteoporosis and osteoporotic fracture, improve bone metabolism, and improve the quality of life of patients with osteoporosis. It is worthy of clinical application. However, there are some limitations in the present study, such as the relatively small number of cases and the short observation time of 2 years, which needs to be extended to 3–5 years for further observation.
